# Supplementary material for: From sequence to enzyme mechanism using multi-label machine learning
Source: BMC Bioinformatics. 2014 May 19;15:150. doi: 10.1186/1471-2105-15-150 (PMC4229970; doi:10.1186/1471-2105-15-150)
Supplement: Additional file 2 — Java code of ml2db. Additional file ml2db_code.tar.gz contains the Java source code to run the multi-label machine learning experiments and save the results to database. The code’s Javadoc is included. [file 1471-2105-15-150-S2.zip › additional file 2/ml2db/ml2db/doc/test/ml2db/weka/package-tree.html]

test.ml2db.weka Class Hierarchy


JavaScript is disabled on your browser.


- Overview
- Package
- Class
- Use
- Tree
- Deprecated
- Index
- Help

- Prev
- Next

- Frames
- No Frames

- All Classes

# Hierarchy For Package test.ml2db.weka

Package Hierarchies:

- All Packages

## Class Hierarchy

- java.lang.Object
  - junit.framework.Assert
    - junit.framework.TestCase (implements junit.framework.Test)
      - test.ml2db.weka.ArffPropsTableTest
      - test.ml2db.weka.ArffTest
      - test.ml2db.weka.AttributeFactoryTest
      - test.ml2db.weka.DataSetCheckerTest
      - test.ml2db.weka.DataSetDbLoaderTest
      - test.ml2db.weka.DataSetGeneratorTest
      - test.ml2db.weka.DataSetManagerTest
      - test.ml2db.weka.DataSetWriterTest
      - test.ml2db.weka.InstancesFillerTest
      - test.ml2db.weka.InstAttValueMapTest
      - test.ml2db.weka.WekaTest

- Overview
- Package
- Class
- Use
- Tree
- Deprecated
- Index
- Help

- Prev
- Next

- Frames
- No Frames

- All Classes
